# Conceptual Mediation in Technomoral Change: Reply to Danaher and Sætra

**DOI:** 10.1007/s10677-025-10481-4

**Published:** 2025-01-27

**Authors:** Jeroen K.G. Hopster, Jon Rueda, Robin Hillenbrink

**Affiliations:** 1 Utrecht University, Utrecht, The Netherlands; 2 Spanish National Research Council, Madrid, Spain; 3 University of Twente, Enschede, The Netherlands

**Keywords:** Technomoral change, Technology, Concepts, Moral theory, Reproductive autonomy, Conceptual mediation, Second-order effects

## Abstract

Philosophers of technology have identified various mechanisms through which technology can change moral norms, values, beliefs and practices. Danaher and Sætra (2023) offer a useful systematization of these mechanisms, with no claim to being exhaustive. We contribute to their work by analyzing how the mediating role of moral concepts fits into this scheme. First, we point out that concepts mediate the moral effects of technological changes, a process we call conceptual mediation. We illustrate this with the concepts of ‘brain death’ and ‘reproductive autonomy’, whose moral implications crystallized in the interplay with new medical technologies. Subsequently, we argue that conceptual mediation is best understood as a type of second-order mediation, which channels the moral implications of the first-order technological mediations identified by Danaher and Sætra (decisional, relational, perceptual). We conclude that second-order mediation plays a central and underappreciated role in processes of technomoral change.

## Introduction

In a recent contribution to this journal, Danaher and Sætra (2023) have systematized the mechanisms that underlie processes of technomoral change. Their taxonomy identifies causal pathways through which technology affects ‘social morality’ – prevalent norms about what actions are deemed valuable, permissible, etc. – by changing human decision options, relations, and perceptions.^1^ As such, their work exemplifies the increasing appreciation in philosophical and ethical work of the mediating role of technology in our moral lives and social institutions (e.g. Verbeek 2008; Benn and Lazar 2022; van de Poel et al. 2023; etc.).

The taxonomy by Danaher and Sætra constitutes a major contribution to systematizing, in a descriptive manner, how technology affects and transforms moral systems. While typologies of technomoral change are not new (e.g. Swierstra and Waelbers 2012; Hopster et al. 2022), their overview is the most comprehensive to date. Yet, as the authors submit, their taxonomy does not aspire to be exhaustive and is open to elaboration and revision. In this article, we propose a friendly amendment that captures a critical feature of technomoral change not thematized in their work, nor in most prior work on the topic: the mediating role of concepts.^2^ We argue that concepts mediate the moral effects that technological changes produce, a process we call *conceptual mediation* (see also Hopster and Löhr 2023; Sect. 4).

By way of initial elaboration on this proposal, we distinguish between three theses about the mediating role of concepts in technomoral change. First, one could defend a **weak thesis**, according to which moral change is frequently mediated by concepts, but this is *not necessarily* the case. Consider an example that Danaher and Sætra discuss: the creation of the smartphone, which – amongst various other implications – has contributed to the expectation of being highly responsive online. One could argue that this new expectation is morally significant, but not conceptually mediated: the change occurs purely at a behavioural level. The weak thesis presupposes a rather liberal understanding of ‘social morality’: changes to social morality include shifts in what a society regards as desirable behaviour, even in the absence of an explicit conceptualization of the corresponding moral norms. 

Second, one could defend a **moderate thesis**, according to which moral change is *necessarily* mediated by concepts. Parting from the weak thesis, examples that *only* involve behavioural changes do not qualify as *moral* changes, properly understood. It is a necessary feature of moral change that it involves changed application conditions of moral concepts, such as ‘duty’, ‘right’, ‘permissible’, ‘just’, etc. For instance, the normative expectation of being highly responsive online might give rise to a correlative ‘duty to respond’. If it does, then this qualifies as a genuine moral change, characterized by the emergence of a new moral duty. Furthermore, it is a conceptually mediated change: at minimum, the articulation of this new moral duty requires scrutiny over the extension of the concept of ‘duty’.

Third, one could defend a **strong thesis**, according to which moral change is not merely *mediated* by morally relevant concepts, but also requires conceptual *change *– i.e., the modification of existing moral concepts, or the emergence of new moral concepts. By way of illustration, consider how property norms might evolve in virtual worlds. The moderate thesis suggests that this will inevitably involve a judgement about appropriate conceptualization: can the concept of ‘ownership’ be extended to objects in virtual worlds? However, the strong thesis makes the additional claim that this does not leave the concept of ownership itself unscathed: by extending it to a new realm, the concept itself also changes. For instance, shifting property norms in this new hybrid domain might give rise to appeals to a new concept of ‘digital ownership’.

Our aim in outlining these three theses is not to provide any of them with a fully fledged defence; doing so goes beyond the ambition of this short paper. Instead, we wish to illustrate that conceptual mediation is a rich topic for philosophical reflection, and highly pertinent to scrutinizing the moral implications of technomoral change. The moderate thesis seems plausible: all technomoral changes, properly understood, involve mediation at the level of moral concepts, which determine their moral implications. But even if the weak thesis could be defended, and conceptual mediation turns out not to be a *necessary* feature of technomoral change, it still plays a frequent role in such change. No more is needed to justify the rationale of the present paper: a thorough understanding of technomoral change requires coming to terms with the mediating role of concepts, which is not addressed in Danaher and Sætra’s otherwise helpful taxonomy.

In Sect. 2 we further illustrate our thesis, by describing two technomoral changes in the field of biomedical ethics that have been mediated by concepts: the case of ‘brain death’ and of ‘reproductive autonomy’. In Sect. 3, we investigate how conceptual mediation can be integrated into Danaher and Sætra’s taxonomy. We argue that changes mediated by concepts are not merely a further *type* of technomoral change, in addition to the three overarching types that Danaher and Sætra identify (decisional, relational, perceptional). Instead, we propose that concepts function as ‘second order mediators’, which channel the moral effects of the first-order technomoral changes Danaher and Sætra highlight. Section 4 concludes that studying second-order moral mediations is a promising avenue to advance future work on technomoral change.

## Brain Death and Reproductive Autonomy

Emerging technologies can affect our general conceptual schemes, as well as specific concepts and conceptions, as pointed out in various works of recent technology ethics (Hopster et al. 2023; Hopster and Löhr 2023; Löhr 2023; Marchiori and Scharp 2024). One much-discussed example is the concept of ‘brain death’ (e.g. Baker 2019; Nickel et al. 2022), which emerged as a fundamentally novel category of thought around the 1960s. In this period, advances in intensive therapies, especially mechanical ventilation and artificial hydration and feeding, led to a reconceptualization of the medical and legal understanding of life and death. These technologies made it possible to keep alive a patient who had irreversibly lost complete brain function, but who might still have active cardiorespiratory function. This new human state led to questioning the cardiorespiratory conception of death. After a pioneering report by a committee from Harvard Medical School (Beecher et al. 1968) the concept of ‘brain death’ gained traction in bioethics. This happened alongside changes in the respective moral norms, rights, and duties. The concept became indispensable to morally justifying new end-of-life practices, such as the permissibility of disconnecting patients from life support measures. The concept also served as a novel moral legitimization to retrieve organs from patients that were neurologically deceased, but still alive on the cardiorespiratory level, and could thus maintain optimal functioning of organs for transplantation. In this way, the concept of brain death was accompanied by the promotion of new moral norms, such as the “dead donor rule”, according to which organ retrieval cannot be the cause of death (a norm that is not without controversy today) (Rodríguez-Arias 2018).

Dahaher and Sætra (2023) also discuss the example of brain death. They do so in the context of decisional moral change: mechanical ventilation technologies changed the available choice options. In essence, the technologies enabled new decision options: keeping someone’s body alive after their brain had ceased functioning, which yielded the further option to preserve organs for donation. However, this characterization, though not mistaken, leaves the moral significance of the change implicit. It fails to thematize how a new and morally significant concept – brain death – became a central reference point for medical codes about the permissibility of organ transplantation, discussions about the permissibility of life extension and euthanasia, and discussions about what a meaningful life or a dignified death amounts to. As we propose in Sect. 3, our more comprehensive taxonomy of technomoral change makes explicit how concepts mediate the moral implications of the new technology.

Now consider a second example, less widely discussed in the literature on technology ethics, which describes an instance of conceptual mediation through the concept of ‘reproductive autonomy’. This concept – sometimes called ‘procreative freedom’ – was popularized in the second half of the 20th century and played a major role in legal constitutional debates about abortion and contraception. The concept of reproductive autonomy gained even more importance in the 1990s, due to the consolidation of assisted reproductive technologies and the advocacy of scholars such as John A. Robertson (Robertson 1994; see Rueda 2024). Until the first birth resulting from in vitro fertilization (IVF) in 1978, human reproduction had been limited to sexual intercourse between fertile opposite-sex couples (Silver 1997; Rueda 2022). Since then, IVF has opened the doors of reproduction to people with fertility problems, same-sex couples, single mothers and fathers, and couples known to be carriers of genetic diseases. The concept of reproductive autonomy mediated moral understandings of the novel choices opened by reproductive and genetic technologies. For instance, in the context of disputes over the moral legitimacy of these technologically mediated reproductive options, reproductive autonomy was propagated as a moral right (Robertson 1994). While the precise scope of reproductive autonomy and its particular embodiment as a moral right remain contested (Johnson 2021; Lee 2022), appeal to this right has nonetheless shaped the bioethical debate and has become a focal point in discussing the moral and legal implications of IVF technology. Nowadays, reproductive autonomy enjoys legal protection in multiple countries; in some countries, such as South Africa, it is even regarded as a constitutional right (Thalder 2023).

Danaher and Sætra do not discuss the example of reproductive autonomy. More importantly for our purposes, they remain silent on the dynamics of conceptual mediation in technomoral change. Sympathizers might think that this is a trivial omission. Technomoral change is a broad phenomenon, and certainly no feasible taxonomy can capture *all* of its features. However, we submit that outlining the role of conceptual mediation makes a non-trivial contribution to understanding the target phenomenon. As both examples indicate, it is often (and arguably even necessarily) the case that technologies exert their transformative effect on morality *through* concepts. Coming to terms with the moral effects of technological change requires engaging with the conceptually mediated principles, rights, and duties that give expression to these effects.

## An Improved Taxonomy of Technomoral Change

**Fig. 1:**
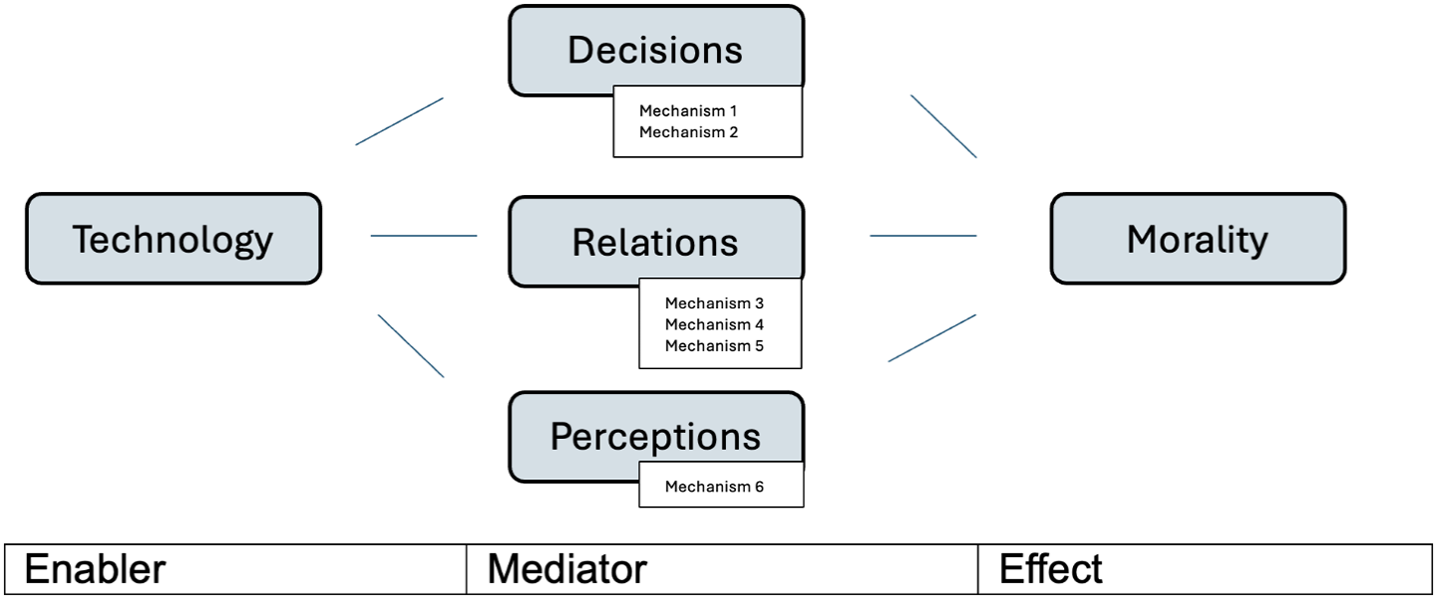
Reconstruction of Danaher and Sætra’s taxonomy of technomoral change

In this section we recast our proposal about conceptual mediation in terms of the technomoral change taxonomy outlined by Danaher and Sætra. According to Danaher and Sætra (2023), there are three general *types* of technomoral change: technology changes morality via decisions, via relations, and via perceptions.^3^ These general types cover six different *mechanisms*, i.e. different causal pathways through which technomoral change of a given type takes place (Fig. 1). Decisional change takes place when technology adds or reduces decision options (mechanism 1), or changes decision-making costs (mechanism 2). Relational change takes place when technology enables new relationships (mechanism 3), changes burdens and expectations in relationships (mechanism 4), or changes the balance of power in society (mechanism 5). Perceptual change takes place when technology gives rise to new information, mental models, and metaphors (mechanisms 6). Although the authors do not spell out the causal role of technology in their framework, it seems plausible to infer that, for any of these mechanisms, technology constitutes an enabling (or constraining) cause that changes affordances in the decisional, relational and perceptual realms.

The final part of their taxonomy identifies the moral effects that these changes bring about. These effects are varied, including raising new dilemmas, generating new moral rules, changing moral rights and duties, changing evaluations, reprioritizing values, expanding the moral circle, etc. Danaher and Sætra note that moral effects can be hard to predict: the same technologies might yield various – and sometimes opposed – moral changes, depending on the broader social and political context in which they are developed and implemented. We might add that moral effects are multiply realizable: the same moral effects can result from different mechanisms of technomoral change.

Where should conceptual mediation fit into this schema? *Prima facie*, one might want to list conceptual mediation as an additional *type* of change, on par with decisional, relational, and perceptual change. However, this proposal is not ideal, since, on closer inspection, conceptual mediation appears deeply intertwined with all the types of technomoral change that Danaher and Sætra identify. For instance, the mechanical ventilator can be discussed as an example of decisional moral change, but it is also an example of conceptually mediated moral change, through the novel concept of ‘brain death’, with associated rights and duties. Similar examples of conceptual mediation can be given in the context of ‘perceptual’ and ‘relational’ changes. But if conceptual mediation is deeply entangled with perceptual, decisional, and relational changes, then it seems inadequate to classify it as a distinct type of technomoral change.

A more appropriate classification, we submit, makes explicit that concepts channel the moral effects of changes in decisions, relations, and perceptions. By this, we mean that the specific conceptualization of the implications of technological changes is co-constitutive of their moral effects. Consider again the case of the mechanical ventilator: it was not only the new affordances that mediated the moral changes that ensued, but also the conceptual framing of these as being cases of ‘brain death’, with associated norms for permissible and impermissible actions that can be undertaken on patients in such a state. The technologically mediated emergence of a new physical state afforded a change in human morality, but it was only through conceptual mediation that this change was subsequently realized.

**Fig. 2:**
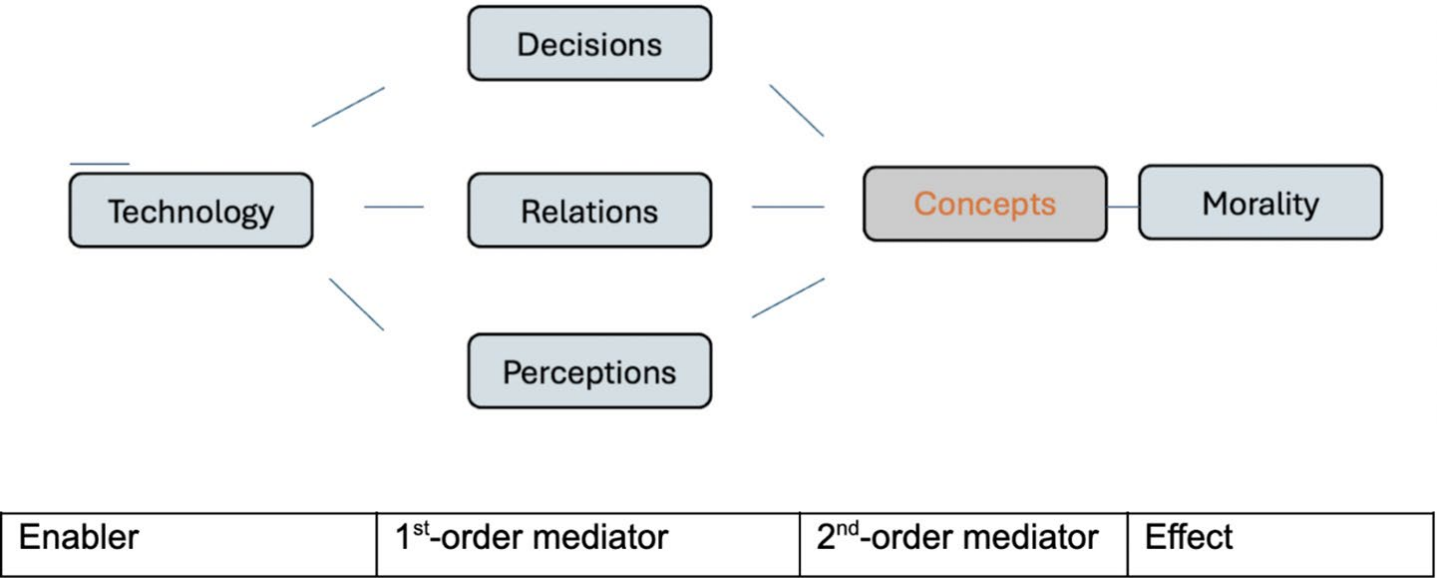
Modified typology of technomoral change

Expanding on this idea, we think of concepts as ‘second-order mediators’, to distinguish them from the first-order mediators that Danaher and Sætra identify (Fig. 2). First-order mediations by technology afford relationships, perceptions, and actions, that are relevant to human cooperation and social behaviour – the constituents of ‘social morality’ in a broad sense. Second-order mediations concern the conceptualizations of these changes in terms of morally relevant concepts, which yield implications for norms about autonomy, consent, fairness, friendship, privacy, trust, and associated rights, duties, etc. – the constituents of ‘moral systems’ in a narrower sense. Hence, concepts serve to institutionalize and entrench technomoral changes: they provide the moral lens through which new technology-induced moral norms, values, beliefs and practices are interpreted and realized.

Concepts both enable and constrain technomoral change. That is to say, concepts are not only mediators of moral *change*. In principle, concepts can also serve the opposite function of *stabilizing* moral systems, in the face of countervailing pressures. Interpreting new affordances through familiar categories of thought can put a brake on moral change; doing so may be conducive to ‘moral conservatism’, rather than ‘moral accelerationism’ (Danaher and Hopster 2022). For instance, it might be argued that the traditional concepts of ‘moral rights’ and ‘moral agency’, whose extension is typically limited to human beings, still apply in the age of intelligent robots and AI systems: non-human entities cannot be regarded as ‘moral agents’ and should not be granted ‘moral rights’. Hence, conceptual mediation should not be equated with moral change: existing concepts might be preserved, thereby facilitating moral stability in the face of technological change.^4^

## Conclusion

**Fig. 3:**
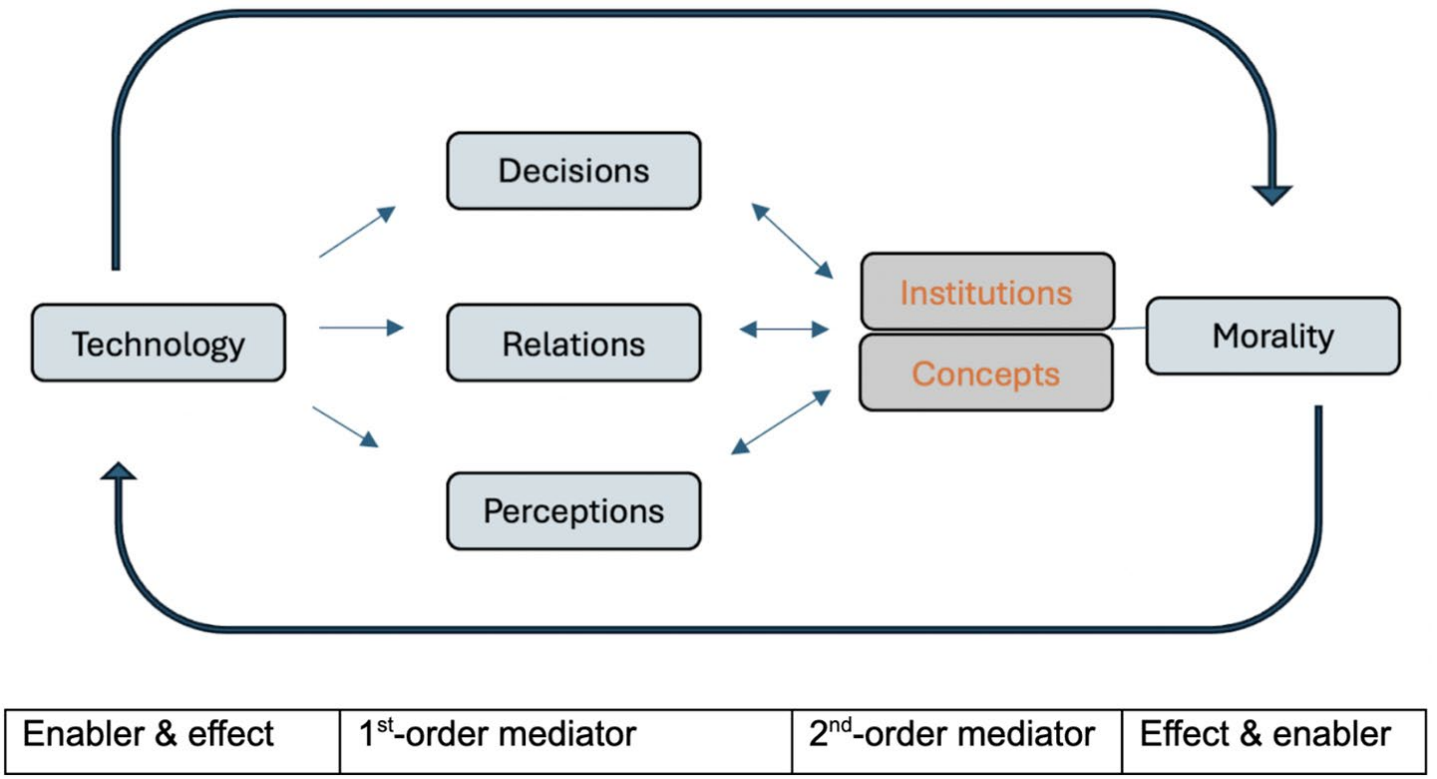
Expanded taxonomy of technomoral change, which highlights the bidirectional dynamics between technology and morality: changes in morality can feed back into the use and design of technologies

We have argued that the taxonomy of technomoral change outlined by Danaher and Sætra can be refined by elucidating the role of conceptual mediation. Concepts structure the moral effects of technological changes, as mediators of both moral stability and change. Are concepts unique in fulfilling this role? The answer depends on whether one adopts a less or more expansive view of what is constitutive of social moral systems. On a broad view, ‘social morality’ also involves the legal, social, and political institutions that structure moral practices: it is *through* institutions that moral effects in society are realized (cf. Hopster and Maas 2024). Assuming this broad view, an even more comprehensive understanding of technomoral change might incorporate institutions as additional second-order mediators, which enable and constrain first-order changes in social morality (Fig. 3). Furthermore, a more comprehensive schematization might account for the feedback relations between them. We can only sketch these relations and dynamics here; fleshing them out in detail strikes us as a promising line of future inquiry.

## Data Availability

N/A.

## Footnotes

1 The concept of technomoral change is sometimes understood to describe bidirectional changes: morality influencing technology and technology influencing morality (Swierstra et al. 2009; see also our discussion in Sect. 3, esp. Figure 3). By contrast, Danaher and Sætra (2023) limit their treatment to the unidirectional influence of technology on social moral systems.

2 More specifically, in this article we focus on moral concepts (thin and thick), as well as concepts that are arguably descriptive, but associated with various moral norms (e.g. our example of ‘brain death’ in Sect. 2), and therefore indirectly with the application of moral concepts.

3 Or, to use our own terminology: decisions, perceptions, and relations are (first-order) *mediators* of technomoral change.

4 Note that this is compatible with the strong thesis, stated in Sect. 1, according to which all instances of moral change require conceptual change.
